# Mexican Hispanics show significant improvement in lung function approximately 1 year after having severe COVID‐19

**DOI:** 10.1113/EP091934

**Published:** 2024-10-24

**Authors:** Arturo Cortes‐Telles, Luis Alberto Solís‐Díaz, Heidegger Mateos‐Toledo, Jordan A. Guenette, Gerald Stanley Zavorsky

**Affiliations:** ^1^ Clínica de Enfermedades Respiratorias, Hospital Regional de Alta Especialidad de la Península de Yucatán, IMSS‐Bienestar Mérida Mexico; ^2^ Clínica de Enfermedades Intersticiales del Pulmón, Instituto Nacional de Enfermedades Respiratorias CdMx Mexico; ^3^ Centre for Heart Lung Innovation, Providence Research The University of British Columbia and St. Paul's Hospital Vancouver Canada; ^4^ Department of Physical Therapy The University of British Columbia Vancouver Canada; ^5^ Department of Physiology and Membrane Biology University of California at Davis Davis California USA

**Keywords:** diffusing capacity, obstruction, recovery, restriction, SARS‐CoV‐2, spirometry

## Abstract

The long‐term effects of COVID‐19 on lung function are not understood, especially for periods extending beyond 1 year after infection. This observational, longitudinal study investigated lung function in Mexican Hispanics who experienced severe COVID‐19, focusing on how the length of recovery affects lung function improvements. At a specialized COVID‐19 follow‐up clinic in Yucatan, Mexico, lung function and symptoms were assessed in patients who had recovered from severe COVID‐19. We used *z*‐scores, and Wilcoxon's signed rank test to analyse changes in lung function over time. Lung function was measured twice in 82 patients: the first and second measurements were taken a median of 94 and 362 days after COVID‐19 diagnosis, respectively. Initially, 61% of patients exhibited at least one of several pulmonary function abnormalities (lower limit of normal = –1.645), which decreased to 22% of patients by 390 days post‐recovery. Considering day‐to‐day variability in lung function, 68% of patients showed improvement by the final visit, while 30% had unchanged lung function from the initial assessment. Computed tomography (CT) scans revealed ground‐glass opacities in 33% of patients. One year after infection, diffusing capacity of the lungs for carbon monoxide *z*‐scores accounted for 30% of the variation in CT fibrosis scores. There was no significant correlation between the length of recovery and improvement in lung function based on *z*‐scores. In conclusion, 22% of patients who recovered from severe COVID‐19 continued to show at least one lung function abnormality 1 year after recovery, indicating a prolonged impact of COVID‐19 on lung health.

## Abstract (highlights)

- **What is the central question of this study?**
  How does the length of recovery from COVID‐19 affect lung function improvements?
- **What is the main finding and its importance?**
  Around one‐fifth of patients who recovered from severe COVID‐19 continued to show at least one lung function abnormality 1 year after recovery, indicating a prolonged impact of COVID‐19 on lung health.

## 1 INTRODUCTION

Patients who have recovered from coronavirus disease 2019 (COVID‐19), caused by severe acute respiratory syndrome coronavirus 2 (SARS‐CoV‐2), frequently encounter persistent health complications and symptoms enduring well beyond the initial 3‐month period post‐infection (van den Borst et al., 2021). Observational studies conducted over 1 year post‐infection reveal that the incidence of abnormal forced vital capacity (FVC) and diffusing capacity of the lungs for carbon monoxide (DLCO) ranged between 2% and 11% and between 7% and 58%, respectively (Chommeloux et al., 2023; Corsi et al., 2022; Zhou et al., 2021). Notwithstanding, a gradual and sustained improvement in pulmonary function post‐COVID occurs, extending at least up to 12 months post‐COVID‐19 infection (Fumagalli et al., 2022).

Even after 2 months post‐infection, ∼15% and ∼55% of individuals demonstrated FVC and DLCO values below 80% of predicted, respectively. Yet, by 12 months post COVID‐19, the proportions of those below 80% of predicted significantly declined to ∼5% and ∼40% (Tarraso et al., 2022). Additionally, mean predicted DLCO and FVC increased from 77% and 92% of predicted at 3 months post‐COVID to 88% and 98% at 12 months, respectively (Wu et al., 2021).

Despite these recuperative trends, ∼40–60% of individuals previously affected by COVID‐19 continue to exhibit symptoms 1 year post‐infection (Bellan, Baricich et al., 2021; Steinbeis et al., 2022; Tarraso et al., 2022; Zhao et al., 2021). Nearly 60% experience varying intensities of dyspnoea (Bellan, Baricich et al., 2021; Steinbeis et al., 2022; Tarraso et al., 2022; Zhao et al., 2021). Those with enduring dyspnoea exhibit distinctly pronounced restrictive patterns on spirometry, reduced DLCO, decreased functional capacity, and lower oxygen saturation levels after physical exertion (Cortes‐Telles et al., 2021; Wong et al., 2021). The reduced lung function post‐COVID has significant clinical implications, especially given the observed increase in mortality rates among survivors 12 months post‐infection (Mainous et al., 2021).

Many studies have used the percentage of predicted value as a metric to assess the recovery of pulmonary function post‐COVID‐19 (Bellan, Soddu et al., 2021; Blanco et al., 2021; Guler et al., 2021; Han et al., 2021; Huang et al., 2020; Liang et al., 2020; Liu et al., 2020; Mo et al., 2020; Qin et al., 2021; Shah et al., 2021; Sonnweber et al., 2021; Zhao et al., 2020). However, this method has been scrutinized as the percentage predicted value at the lower limit of normal (LLN) decreases with age, starting at about 40 years of age up to death (Quanjer et al., 2012; Zavorsky & Cao, 2022). Notably, in a recent study, about 15% of post‐COVID‐19 patients were inaccurately categorized as having mild diffusion impairment when utilizing a threshold of less than 80% of predicted rather than a *z*‐score of less than –1.645 (Cortes‐Telles et al., 2022). The adoption of *z*‐scores, representing either the 5th percentile (*z* = –1.645) or the 2.5th percentile (*z* = –1.96), avoids the issue of a reduced percentage predicted at the LLN with advancing age. The most recent European Respiratory Society (ERS)/American Thoracic Society (ATS) interpretive strategies has advocated using *z*‐scores instead of percentage of predicted (Stanojevic et al., 2022).

The change in pulmonary function abnormalities in those previously infected with COVID‐19 is not well studied, particularly changes in *z*‐scores over time. Longer recovery times may allow for the resolutions of inflammation and repair of lung tissue. Over time, these pathological changes can partially reverse as inflammation subsides and the body's natural healing processes, including remodelling of lung tissue and resolution of fibrosis, take place (Fraser et al., 2020). As such, this study aimed to evaluate lung function among Mexican Hispanic patients who had severe COVID‐19 and its recovery. We hypothesized that patients with a longer recovery time between COVID‐19 diagnosis and pulmonary function testing would have improved pulmonary function compared to those tested earlier.

## 2 METHODS

### 2.1 Ethical approval

The Ethics Committee of the Hospital Regional de Alta Especialidad de la Península de Yucatán – IMSS Bienestar, Mérida, Mexico approved this study (Protocol number 2023‐003), which was properly registered in accordance with Clause 35 of the *Declaration of Helsinki*. Upon admission, every patient signed an informed consent to receive all treatment, including follow‐up.

The primary outcome of the study was to measure changes in lung function over time using *z*‐scores during follow‐up. Secondary outcomes included assessing the correlation between chest computed tomography (CT) scan findings and abnormalities in pulmonary function tests, determining the relationship between improvements in lung function tests and symptom improvement, and establishing whether there was an association between the presence of comorbidities and lung function recovery.

### 2.2 Patients

This observational longitudinal study was conducted at the long‐term follow‐up COVID‐19 Clinic at the Hospital Regional de Alta Especialidad de la Península de Yucatán – IMSS Bienestar in Mérida, Mexico from March 2021 to August 2021. We consecutively enrolled 100 patients hospitalized during this period. Inclusion criteria were adults over 18 years old recovering from severe COVID‐19. Severe COVID‐19 in adults is defined by the World Health Organization as any of the following criteria: oxygen saturation below 90% on room air; severe pneumonia; or signs of severe respiratory distress, such as the use of accessory muscles, inability to complete full sentences, or a respiratory rate exceeding 30 breaths per minute (WHO, 2023). Exclusion criteria included patients with pneumonia from causes other than SARS‐CoV‐2 infection, patients confirmed with mild or moderate COVID‐19, and patients with only one evaluation during follow‐up. All patients were scheduled for pulmonary function testing approximately 1, 3, 6 and 12 months after COVID‐19 diagnosis. Height and weight were recorded using a mechanical weigh beam scale equipped with a height rod. Body mass index (BMI) was calculated by dividing the weight in kilograms by the square of the height in metres (kg/m^2^).

### 2.3 Evaluation of pulmonary function abnormalities

There were seven pulmonary ailments that we assessed and identified based on the 2022 ERS/ATS interpretation strategies (Stanojevic et al., 2022): (i) restrictive spirometry pattern (forced expiratory volume in 1 s (FEV_1_)/FVC > LLN, and FVC < LLN; (ii) airflow obstruction (FEV_1_/FVC < LLN and FVC > LLN); (iii) mixed disorder (FEV_1_/FVC < LLN and FVC < LLN); (iv) loss of alveolar capillary structure with loss of lung volume (DLCO < LLN, and alveolar volume (*V*_A_) < LLN, and the rate of CO uptake from alveolar gas (*K*_CO_) < ULN); (v) localized loss of lung volume or incomplete lung expansion (failure to take a deep breath or neuromuscular dysfunction), (DLCO < LLN and *V*_A_ < LLN, and *K*_CO_ > ULN); (vi) pulmonary vascular abnormality (DLCO < LLN and *V*_A_ normal); and (vii) alveolar haemorrhage, polycythaemia, increased blood flow (left to right shunt, or post‐exercise; DLCO > ULN). In addition, there was an eighth pulmonary condition that we assessed, but it was not a part of the ERS/ATS interpretation strategy for spirometry; it was those with a preserved FEV_1_/FVC ratio but impaired spirometry (PRISm) (FEV_1_/FVC ≥ LLN and FEV_1_ < LLN). For patients who underwent pulmonary function testing on more than two different occasions, we selected the two post‐COVID‐19 testing dates that were furthest apart.

At each visit patients were asked for presence or absence of symptoms at the time of the visit, including fatigue, shortness of breath on effort, cough, chest tightness, chest pain, sore throat, blocked and/or runny nose, loss of smell, loss of taste, diarrhoea, abdominal pain, muscle or joint pain, headache, tachycardia, sore or red eyes, excessive sweating (over a 24 h period, including night sweats), hair loss and weight loss.

### 2.4 Assessment of lung fibrosis using high resolution computed tomography

A CT scan of the chest was requested at the 12‐month visit, and the time between the onset of the acute illness and the day it was performed was recorded. In patients who underwent a high resolution CT (HRCT) scan at their final visit, the extent of fibrosis was assessed. A simple staging system divided patients based on HRCT results (Goh et al., 2008). HRCT images were scored at five anatomical levels: (i) origin of the great vessels, (ii) main carina, (iii) pulmonary venous confluence, (iv) halfway between the third and fifth sections, and (v) immediately above the right hemi‐diaphragm.

The primary HRCT variable examined was the coarseness of reticular disease, defined as the thickness and visibility of reticular patterns. The severity of reticulation (fibrosis) was scored as follows: grade 0: ground glass attenuation alone; grade 1: fine intralobular fibrosis; grade 2: microcystic honeycombing (air spaces ≤ 4 mm in diameter); and grade 3: microcystic honeycombing (air spaces > 4 mm in diameter). The total coarseness (fibrosis) score was the sum of the scores for all five levels, ranging from 0 to 15. For patients with no disease in one or more CT sections, the coarseness score was adjusted to a five‐level score. For example, if HRCT appearances were normal in one section, a coarseness score of 8 was adjusted to 10 by multiplying by 5/4 (Goh et al., 2008).

### 2.5 Statistical analyses

This study applies current ATS/ERS recommendations (Stanojevic et al., 2022) by using *z*‐scores to rigorously evaluate the persistence and recovery of pulmonary abnormalities in the Mexican Hispanic population. The use of *z*‐scores for pulmonary function test interpretation is more appropriate than percentage predicted values, as the LLN of the percentage predicted changes with age (Quanjer et al., 2012; Zavorsky & Cao, 2022).

A sample size calculation was not conducted, as this was an exploratory data analysis. *z*‐scores for FEV_1_, FVC and FEV_1_/FVC were calculated using the Global Lung Function Initiative (GLI) reference equations for all races (Bowerman et al., 2023), while *z*‐scores for DLCO, *V*_A_, and *K*_CO_ were derived using reference equations elsewhere (Gochicoa‐Rangel et al., 2024). Any value below the LLN (5th percentile, *z*‐scores < –1.645) were considered abnormal. Changes in pulmonary function indices between the initial and final visits were analysed using Student's paired *t*‐test for normally distributed *z*‐scores. The Shapiro–Wilk test was used to verify normality (Ghasemi & Zahediasl, 2012). When the *z*‐scores were not normally distributed, Wilcoxon's signed‐rank test was applied. It is noted, however, that in samples >40, violations of normality may not pose a significant issue, allowing for the use of parametric methods (Ghasemi & Zahediasl, 2012). Additionally, changes in the proportion of participants with normal spirometry, diffusing capacity, or both, between the initial and final visits, were assessed using McNemar's test with continuity correction. Similar methods were used to compare the proportion of participants with various lung abnormalities across the two visits. To account for multiple comparisons and control the false discovery rate, the Benjamini–Hochberg procedure was applied (Benjamini & Yekutieli, 2001).

Overall changes in lung function at each visit were assessed by summing the *z*‐scores for FEV_1_, FVC, FEV_1_/FVC, DLCO, alveolar volume (*V*_A_) and the rate of CO uptake from alveolar gas (*K*_CO_). A 95% confidence interval (CI) for these changes was determined using 1000 bootstrapped samples. Bootstrapping methods, which do not assume a specific distribution, provided a more robust estimation of the mean difference for non‐normally distributed data.

To investigate the relationship between the improvement in overall summed *z*‐scores and the time interval between the initial and final lung function tests, a linear regression analysis was conducted. The change in summed *z*‐scores (*y*‐axis) was plotted against the number of days between the initial and final tests. The model's fit was evaluated by examining standardized residuals against standardized predicted values to assess linearity, homoscedasticity and normality of residuals. Furthermore, an analysis of covariance was used to examine differences in the improvement in *z*‐scores between men and women, controlling for the initial summed *z*‐score value.

A binary logistic regression was performed to identify whether variables such as sex, age, BMI, number of pre‐existing risk factors for cardiovascular disease (morbid obesity (BMI ≥ 40/kg/m^2^), self‐reported hypertension, self‐reported diabetes, self‐reported current or previous (within previous 6 months) smoker), number of days between initial and final pulmonary function test (PFT), or the change in symptomatology were associated with a meaningful change in summed *z*‐scores (1 = meaningful change; 0 = no meaningful change). The influence of the initial summed *z*‐scores from the PFT were also taken into consideration for affecting outcome. The criteria for meaningful change in summed *z*‐scores is outlined in Appendix A. The total number of persistent symptoms at both the initial and final visits was compared using Wilcoxon's signed‐rank test, and the association between changes in symptom count and summed *z*‐scores was assessed using Spearman's rank correlation coefficient.

To explore the relationship between fibrosis and DLCO, the Goh fibrosis score (ranging from 0 to 8) was correlated with the DLCO *z*‐scores. The same radiologist evaluated the entire set of imaging data to maintain consistency.

All figures were created using GraphPad Prism (version 10.3.0.507, GraphPad Software, Boston, MA, USA), and statistical analyses were performed using IBM SPSS Statistics (Version 29.0.1.0; IBM Corp., Armonk, NY, USA) and RStudio (Version 2024.04.2, build 764). Statistical significance was set at *P* < 0.05.

## 3 RESULTS

### 3.1 Baseline characteristics

A total of 100 patients were recruited, but 18 patients were lost at follow‐up. This left 82 patients who had pulmonary function evaluated on two different occasions after being afflicted with severe COVID‐19 are presented in Table 1. There were 33 females with the following anthropometric characteristics at the first measurement: mean (SD) age 50 (13) years; weight 74 (14) kg; height 146 (6) cm; BMI 34.5 (5.9) kg/m^2^. There were 49 males with the following anthropometric characteristics at the first measurement: mean (SD) age 48 (13) years; weight 80 (18) kg; height 160 (7) cm; BMI 31.2 (6.6) kg/m^2^. Thirty and 21 patients self‐reported hypertension and diabetes, respectively. Sixteen patients were former (within 6 months) or are current smokers. Thirteen patients were morbidly obese (BMI ≥ 40 kg/m^2^).

**TABLE 1:** Prevalence of normal and abnormal pulmonary function at the initial and final visit.

	Initial visit	Final visit	Mean difference in proportions [95% bootstrapped CI]
LLN is defined as the 5th percentile (*z*‐score = –1.645)			
Normal spirometry	52% (43/82)	85% (70/82)	33% [23 to 44%]^*^
Normal DLCO	59% (48/82)	87% (71/82)	28% [17 to 39%]^*^
Restrictive spirometry pattern	46% (38/82)	13% (11/82)	−33% [−43 to −22%]^*^
PRISm	40% (33/82)	9% (7/82)	−32% [−43 to −22%]^*^
Airflow obstruction	1% (1/82)	1% (1/82)	0% [–3 to 3%]
Possible mixed disorder	0% (0/82)	0% (0/82)	0% [–3 to 3%]
Loss of alveolar capillary structure with loss of lung volume	35% (29/82)	10% (8/82)	−26% [−37 to −16%]^*^
Localized loss of lung volume or incomplete lung expansion (failure to take a deep breath, or neuromuscular dysfunction)	2% (2/82)	1% (1/82)	−1% [−2 to 6%]
Pulmonary vascular abnormality	2% (2/82)	0% (0/82)	0% [–3 to 3%]
Alveolar haemorrhage, polycythaemia, or increased blood flow (left‐to‐right‐shunt, or post‐exercise)	1% (1/82)	2% (2/82)	1% [0 to 6%]
No. of patients with at least one abnormality	61%(50/82)	22% (18/82)	−39% [−50 to −28%]^*^
No. of patients with normal spirometry and DLCO	39% (32/82)	78% (64/82)	39% [28 to 50%]^*^
LLN defined as the 2.5th percentile (*z*‐score = – 1.96)			
Normal spirometry	62% (51/82)	89% (73/82)	27% [17 to 39%]^*^
Normal DLCO	67% (55/82)	90% (74/82)	23% [15 to 32%]^*^
Restrictive spirometry pattern	38% (31/82)	11% (8/82)	−27% [−38 to −17%]^*^
PRISm	29% (24/82)	7% (6/82)	−22% [−32 to −13%]^*^
Airflow obstruction	0% (0/82)	0% (0/82)	0% [–3 to 3%]
Possible mixed disorder	0% (0/82)	0% (0/82)	0% [–3 to 3%]
Loss of alveolar capillary structure with loss of lung volume	28% (23/82)	7% (6/82)	−21% [−29 to −11%]^*^
Localized loss of lung volume or incomplete lung expansion (failure to take a deep breath, or neuromuscular dysfunction)	1% (1/82)	1% (1/82)	0% [–3 to 3%]
Pulmonary vascular abnormality	2% (2/82)	0% (0/82)	−2% [−7 to 2%]
Alveolar haemorrhage, polycythaemia, or increased blood flow (left‐to‐right‐shunt, or post‐exercise)	1% (1/82)	1% (1/82)	0% [–3 to 3%]
No. of patients with at least one abnormality	46% (38/82)	20%(16/82)	−27% [−39 to −13%]^*^
No. of patients with normal spirometry and DLCO	54% (44/82)	80% (66/82)	27%% [13 to 39%]^*^

*Note*: Abnormal spirometry and DLCO was defined according to the 2022 ATS/ERS technical standards (Stanojevic et al., 2022) using GLI Global equations (Bowerman et al., 2023).

* After correcting for the false discovery rate, there was statistical significance between the two visits (*P* < 0.05). The initial visit was 119 (SD 70) days after COVID‐19 diagnosis [range = 55–367 days]. The final visit was 390 (SD 146) days after COVID‐19 diagnosis [range = 179–724 days].

The first pulmonary function evaluation (i.e., baseline) was conducted at a median of 94 days after severe COVID‐19 infection, with a range from 55 to 367 days. The second evaluation took place at a median of 362 days post‐infection, ranging from 179 to 724 days. For 19 patients, the second evaluation occurred between 502 and 724 days after diagnosis (median = 641 days). The median interval between the two pulmonary function evaluations was 250 days, ranging from 67 to 637 days. Nine patients had intervals between 531 and 637 days (median = 586 days).

Approximately 40% of patients had a combination of normal spirometry + normal DLCO at the initial visit (baseline), which increased to 78% 1 year after COVID‐19 (LLN < –1.645 *z*‐score units) (Table 1). Among those with abnormal spirometry at the initial evaluation, nearly all exhibited a restrictive spirometry pattern. At the initial visit, 46% of patients had a spirometric abnormality, 40% had a pulmonary diffusion abnormality, and about 27% had both a spirometric abnormality and a pulmonary diffusing capacity (D, E, F or G) abnormality. At 1‐year follow‐up, only six patients (7%), had a combination of abnormal spirometry + abnormal DLCO. The same number of variables were statistically significant whether the false discovery rate was controlled for or not (Table 1)

### 3.2 Lung function changes over time

**FIGURE 1:**
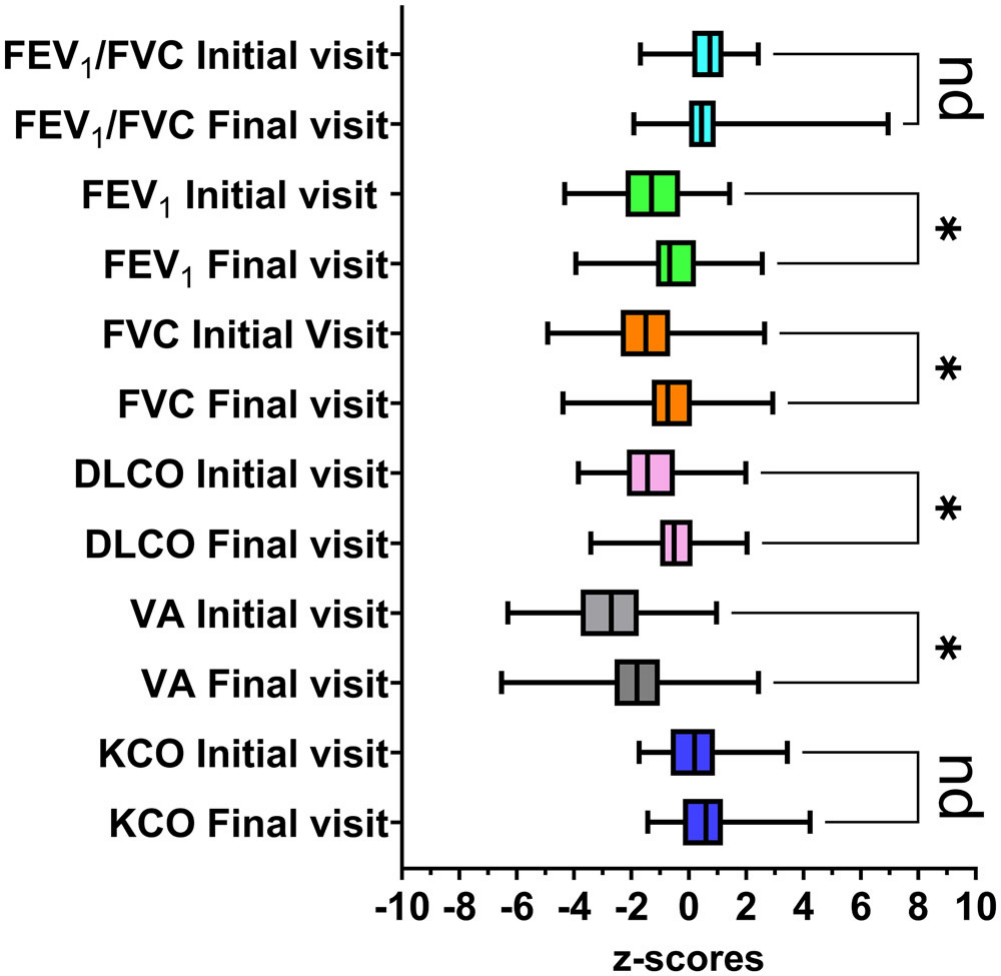
Changes in pulmonary function indices between the initial and final visit controlling for the false discovery rate. The differences in *z*‐scores for each variable were used to determine significant changes. FEV_1_, FVC, DLCO and *V*_A_ improved between visits (*P = *0.0043, *P* = 0.0053 and *P* = 0.0013, respectively) while FEV_1_/FVC ratio and *K*_CO_ did not (nd = not a discovery, *P* = 0.712 and *P* = 0.124, respectively). Mean *z*‐scores (±SD of *z*‐scores) were as follows: FEV_1_ (initial visit) = −1.29 ± 1.24, FEV_1_ (final visit) = −0.46 ± 1.17, FVC (initial visit) = −1.52 ± 1.35, FVC (final visit) = −0.63 ± 1.25, DLCO (initial visit) = −1.37 ± 1.09, DLCO (final visit) = −0.50 ± 1.04, *V*_A_ (initial visit) = −2.79 ± 1.46, *V*_A_ (final visit) = −1.78 ± 1.61. The initial visit occurred at a median of 94 days after COVID‐19 diagnosis (range = 55–367 days). The final visit occurred at a median of 362 days after COVID‐19 diagnosis (range = 179–724 days). The median number of days between visits was 250 days (range = 67–637 days) (82 subjects).

The differences in *z*‐scores for each pulmonary function variable were used to determine significant changes between the two visits (Figure 1). FEV_1_, FVC, DLCO and *V*_A_ improved between visits (*P = *0.0043, *P* = 0.0053 and *P* = 0.0013, respectively) while FEV_1_/FVC ratio, and *K*_CO_ did not (nd (not a discovery), *P* = 0.712 and *P* = 0.124, respectively). Mean *z*‐scores (±SD) were as follows: baseline FEV_1_ = –1.29 ± 1.24, follow‐up FEV_1_ = –0.50 ± 1.04; baseline FVC = –1.52 ± 1.35, follow‐up FVC = –0.63 ± 1.25; baseline DLCO = –1.37 ± 1.09, follow‐up DLCO = –0.50 ± 1.04; baseline *V*_A_ = –2.79 ± 1.46, follow‐up *V*_A_ = –1.78 ± 1.61.

**FIGURE 2:**
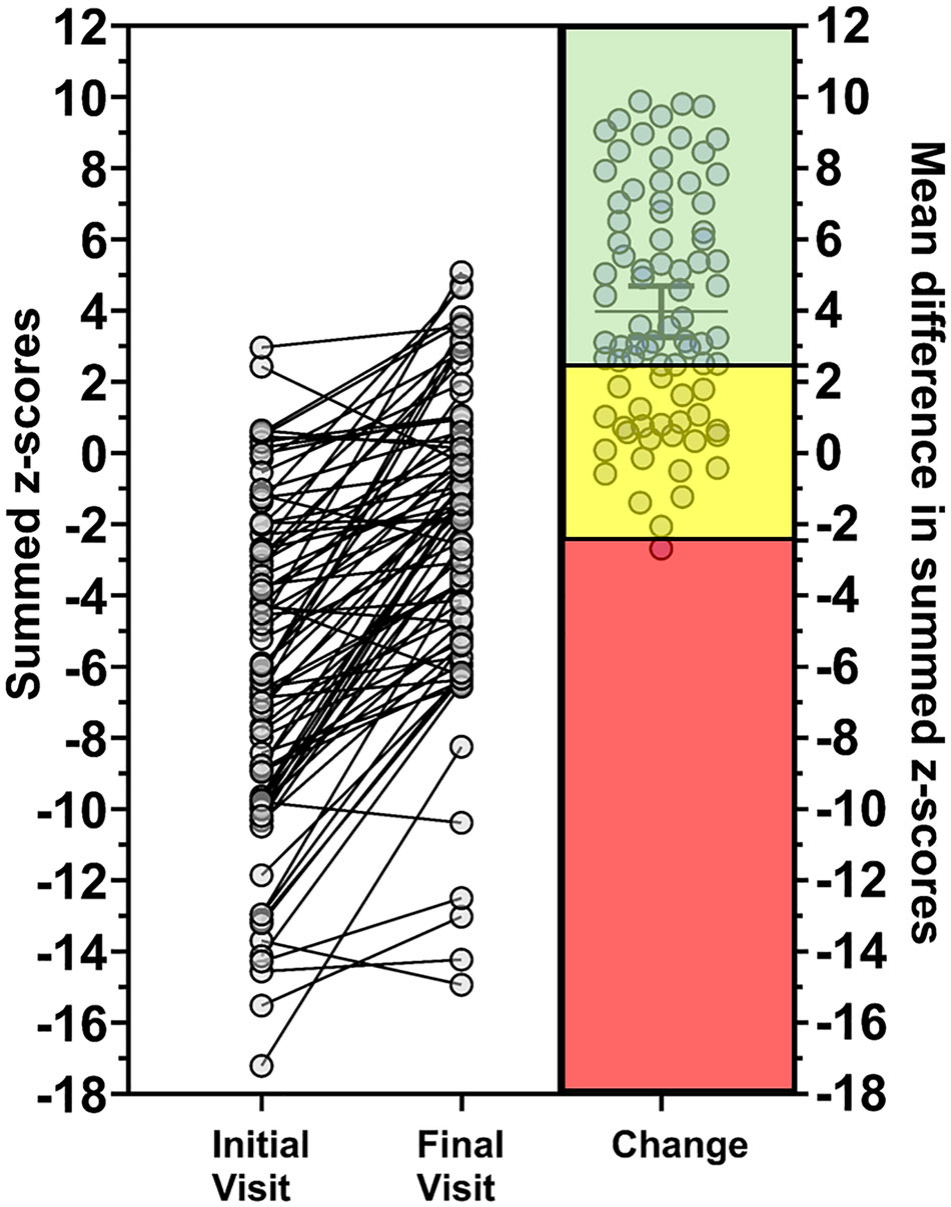
Analysis of summed *z*‐scores in patients post‐COVID‐19 diagnosis. Summed *z*‐scores, which include the FEV_1_/FVC ratio, FEV1, FVC, DLCO, *V*_A_ and *K*_CO_, were computed for each patient at their initial and final visits. The baseline (initial visit) summed median *z*‐scores were –6.26 (range = –17.22 to 2.96), and the follow‐up (final visit) median summed *z*‐scores were –1.55 (range = –14.93 to 5.08). There was a median improvement in summed *z*‐scores of +3.19 units with a 95% bootstrapped CI of +2.63 to +5.12 units (Wilcoxon's signed rank test, *Z* = –7.316, *P* < 0.0001). The effect size of this change was +0.89 (95% CI with Hedges's correction = 0.68–1.10). The threshold for a meaningful change in summed *z*‐scores was set at ±2.23 units, which accounts for the day‐to‐day variability observed in lung function tests. Among the 82 patients, 56 (68%) showed clinically significant improvements by exceeding a change of more than +2.23 *z*‐score units (the change is inside the green filled area). The other 26 patients saw changes within ±2.23 units, indicating no significant improvement or deterioration (the change is inside the yellow filled area). One patient had a worsening of lung function (the change is inside the red filled area). Additional details on these calculations can be found in Appendix A. The timeline from COVID‐19 diagnosis to the initial visit averaged 119 days (SD = 70; range 55–367 days), while the final visit occurred at an average of 390 days post‐diagnosis (SD = 146; range 179–724 days). The period between the two visits averaged 271 days (SD = 141; range 67–637 days) across the cohort.

The summed *z*‐scores for each patient (initial + final visit), versus the change in summed *z*‐scores between visits are presented in Figure 2. Summed *z*‐scores included the summed *z*‐scores of the FEV_1_/FVC ratio, FEV_1_, FVC, DLCO, *V*_A_ and *K*_CO_. The baseline (initial visit) summed median *z*‐scores were –6.26 (range = –17.22 to 2.96), and the follow‐up (final visit) median summed *z*‐scores were –1.55 (range = –14.93 to 5.08). There was a median improvement in summed *z*‐scores of +3.19 units with a 95% bootstrapped CI of +2.66 to +5.12 units (Wilcoxon's signed rank test, *Z* = –7.316, *P* < 0.0001). The effect size of this change was +0.89 (95% CI with Hedges's correction = 0.68–1.10). Men had a larger improvement in summed *z*‐scores than women (median improvement was +2.45 higher *z*‐score units more than women (95% bootstrapped CI, +0.32 to +4.45 higher summed *z*‐scores in men compared to women, *P *= 0.011); but this was largely due to the lower initial summed *z*‐scores in men (median initial summed *z*‐score = –7.00) compared to women (median initial summed *z*‐score = –4.18). Specifically, for men, the effect of the initial summed *z*‐score on the final *z*‐score was 0.37 *z*‐score units larger than for women and this interaction was statistically significant (*P* = 0.0174), meaning that the relationship between the baseline and final *z*‐scores was stronger for men than for women.

There was a reduction in the number of persistent symptoms between the initial and final visit (median number of symptoms = 4 at the initial visit, versus 3 at the final visit, Wilcoxon's signed rank test, *Z* = –2.01, *P* = 0.044). There was no one symptom that was consistently reduced. When comparing the overall change in symptomatology to the change in summed *z*‐scores, the association was not significant (*P *= 0.066).

Binary logistic regression revealed that being male increased the odds of an improvement in overall *z*‐scores between the initial and final visits by about three‐fold compared to females (odds ratio = 3.2, 95% CI = 1.1–10.0, *P* = 0.033). However, age, BMI, total number of pre‐existing conditions, the number of days between baseline and final PFTs, and changes in symptomatology were not significant predictors. The model explained approximately 9–14% of the variability in whether a ‘meaningful change’ occurred in summed *z*‐scores. The *R* ^2^ values indicate that the model has some explanatory power, but it could likely be improved with additional or more relevant predictors.

When the initial summed *z*‐scores from the first PFT were included in the binary logistic regression model, the sex factor became non‐significant. Instead, higher initial summed *z*‐scores were associated with ∼20% lower odds of experiencing a ‘meaningful change’ in summed *z*‐scores (95% CI = 8%–31%, *P* = 0.0037). With the inclusion of initial summed PFT *z*‐scores, the model explained approximately 20–30% of the variability in whether a ‘meaningful change’ occurred.

### 3.3 Lung function trajectories

The smallest measurable change in summed *z*‐scores was calculated to be ±2.23 units (see Appendix A for details on this calculation). By the final visit, 56 out of 82 subjects (68%) showed an overall improvement in pulmonary function, as indicated by their summed *z*‐scores exceeding +2.23 units (green transparent background in Figure 2). Only one patient experienced a decline greater than –2.23 units (red transparent background in Figure 2). Consequently, two‐thirds of patients exhibited improved overall pulmonary function between the initial and final visits, 30% of patients had no change in pulmonary function (yellow transparent background in Figure 2), and 1% of patients showed worsened pulmonary function.

**FIGURE 3:**
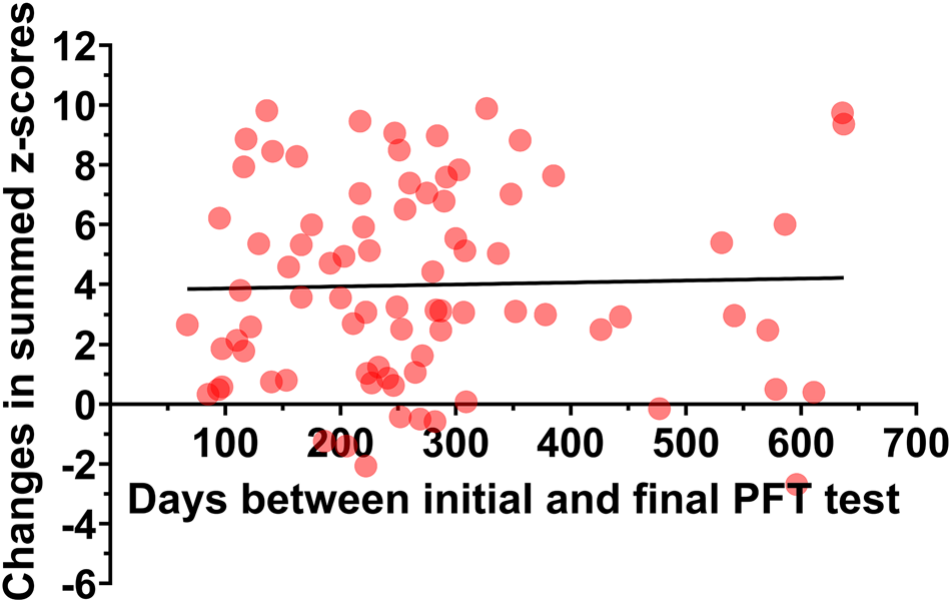
The association between the number of days between the two visits and changes in summed *z*‐scores. No association was present (*r* = 0.028, *P* = 0.802), even when controlling for the number of days since COVID‐19 diagnosis in the initial visit (*r* = 0.000, *P* = 0.979). There was no violation of the key assumptions (homoscedasticity, linearity, normal distribution of residuals).

The association between the number of days between the two measurements and changes in summed *z*‐scores is presented in Figure 3. No association was present (*r* = 0.028, *P* = 0.802), even when controlling for the number of days since COVID‐19 diagnosis in the initial visit (*r* = 0.000, *P* = 0.979). There was no violation of the key assumptions (homoscedasticity, linearity, normal distribution of residuals).

### 3.4 Lung function correlations with CT‐scan images

Among individuals who underwent a HRCT scan near the time of their pulmonary function test (PFT) measurement, there was a moderate negative correlation between the Goh fibrosis score (ranging from 0 to 8) and DLCO *z*‐scores (ranging from –2.74 to +1.09). The correlation was *r* = –0.54 (95% bootstrapped CI = –0.74 to –0.27, *P* = 0.0002, patients), indicating that about 30% of the variance in the extent of fibrosis is shared with DLCO *z*‐scores. Specifically, the regression equation was the following: Fibrosis score = 0.56–1.492 **×** (DLCO *z*‐score), *R* ^2^ = 0.29, standard error of the estimate (SEE) = 2.04, and the 95% CI for the slope ranged from –2.22 to –0.77. Thus, for every 1 unit increase in the fibrosis score, the DLCO *z*‐score decreased by 0.77–2.22 *z*‐score units. Yet, neither the Goh fibrosis score nor the DLCO *z*‐score was correlated with the number of days since the COVID‐19 diagnosis. It is noteworthy that the median length of time between PFT and CT scanning was 38 days (range –106 to +258 days). For 40 of the 44 scans, the HRCT scans occurred nearest to the final PFT, while for four of the 44 scans, the HRCT scans occurred nearest to the first PFT.

## 4 DISCUSSION

The purpose of this study was to examine pulmonary function improvement over time in Mexican Hispanic patients previously afflicted with severe COVID‐19. We observed a significant improvement in pulmonary function approximately 1 year following diagnosis. Using a *z*‐score threshold of –1.645 to define pulmonary function abnormalities, our key findings include the following: (1) at the first measurement, an equal number of patients exhibited either pulmonary diffusion abnormalities or spirometry abnormalities, with 27% having both; (2) the proportion of patients with either abnormal spirometry or abnormal DLCO (or both) was 61% at approximately 94 days post‐diagnosis, which dropped to 22% by the 392 days post‐diagnosis, with 19 patients measured at a median of 641 days post‐diagnosis; (3) considering the day‐to‐day variation in spirometry and diffusing capacity measurements, 68% of patients had improved pulmonary function per summed *z*‐scores between the initial and final visit; (4) there was no association between the number of days between the two visits and changes in summed *z*‐scores, even when controlling for the number of days since the COVID‐19 diagnosis at the first measurement; and (5) 30% of the variation in the extent of fibrosis was associated with DLCO *z*‐scores.

With increasing severity of COVID‐19, the proportion of patients with DLCO below the LLN also increases, especially among those requiring mechanical ventilation compared to those who do not (Abdallah et al., 2021; Cortes‐Telles et al., 2022, 2021; Gochicoa‐Rangel et al., 2021; Morin et al., 2021; van den Borst et al., 2021). When DLCO plus one or more spirometric variable (FEV_1_, FVC or FEV_1_/FVC) has a *z*‐score more negative than –1.645, that would classify as impaired pulmonary function.

In at least 50% of patients with severe COVID‐19 or those who required invasive mechanical ventilation, pulmonary function remained impaired at 90–120 days post‐diagnosis (Ekbom et al., 2021; Hellemons et al., 2022; Konsberg et al., 2023; Morin et al., 2021). Our findings similarly show 61% of our patient cohort had at least one pulmonary function abnormality 120 days post‐COVID‐19 diagnosis when LLN was defined as –1.645 *z*‐score units. These abnormalities can be explained by the histopathological changes described in autopsy studies, primarily characterized by diffuse alveolar damage, initially with high levels of inflammation, which can gradually reverse or evolve into interstitial fibrosis with remodelling, as well as thrombosis and haemorrhage (Angeles Montero‐Fernandez & Pardo‐Garcia, 2021). Thus, the novelty of our study lies in the detailed presentation of the pulmonary function abnormalities found from spirometry and diffusing capacity measurements as well as taking into consideration the day‐to‐day‐variability of spirometry and diffusing capacity. The daily variability in pulmonary function is a critical factor, encompassing physiological fluctuations, the consistency of patient effort during spirometry and diffusion capacity tests, and the precision of the measuring equipment. Our unique approach involves quantifying this variability in terms of z‐scores, enhancing the interpretability and robustness of our findings. The *z*‐score allows for more accurate patient classification, and can provide prognostic information (Brems et al., 2024), so its utilization is imperative for study interpretation.

Longer recovery times were hypothesized to facilitate the resolution of inflammation and the repair of lung tissue. Severe COVID‐19 is frequently linked with significant inflammation and lung parenchymal damage, including diffuse alveolar damage, fibrosis and microvascular injury (Angeles Montero‐Fernandez & Pardo‐Garcia, 2021). Over time, these pathological changes are expected to partially reverse as inflammation decreases and the body's natural healing mechanisms, such as lung tissue remodelling and fibrosis resolution, occur (Fraser et al., 2020). Additionally, extended recovery periods may allow for a reduction in fibrotic changes, as observed in HRCT scans. Evidence suggests that while fibrosis is a significant early outcome in severe COVID‐19 cases, it can diminish in severity over time (Wu et al., 2022). However, our study found no significant association between the interval duration between two pulmonary function evaluations and changes in summed *z*‐scores, even after adjusting for the time elapsed since the initial COVID‐19 diagnosis (Figure 3). Thus, the recovery of spirometry and diffusing capacity is not necessarily dependent on recovery time, but it is individual‐dependent, with some individuals returning to normal pulmonary function faster than others. Nevertheless, we identified a moderate negative correlation between fibrosis scores from HRCT scans and DLCO *z*‐scores, suggesting that a reduction in fibrosis is associated with improved diffusing capacity (*r* = –0.54, *P* = 0.0002). Other studies have shown similar associations between fibrosis scores from CT scans and DLCO (Fraser et al., 2020; Wu et al., 2022).

Recovery of lung function post‐COVID‐19 is likely influenced by multiple complex and interacting factors, making it difficult to isolate the impact of recovery time alone. Factors such as fibrosis, ongoing inflammation and changes in lung mechanics might play significant roles independent of the individual variability. Patients with pre‐existing respiratory conditions such as asthma or COPD, cardiovascular disease, or metabolic disorders like diabetes may experience slower or incomplete lung recovery, as these conditions could complicate post‐infection healing. However, we found that the total number of pre‐existing risk factors did not predict improvement in summed *z*‐scores. The severity of the initial illness could also play a role; yet in this study, the patients were relatively homogeneous as they were all classified as having severe COVID‐19. Demographic and genetic factors, including age, sex and genetic predisposition, could also affect recovery, with older patients likely experiencing slower recovery due to reduced regenerative capacity. In this study, men had a statistically larger improvement in overall *z*‐scores that women (*P* = 0.014), but it was not due to a younger age, as there was no association between age and the change in *z*‐scores. Yet, when the summed *z*‐scores from the initial PFT were taken into consideration, the differences between the sexes were minimized.

Furthermore, various factors between the initial and final tests, such as treatments received, changes in lifestyle such as physical activity levels or exposure to environmental pollutants, or new health issues, could influence pulmonary function independently of the time since COVID‐19 diagnosis. These intervening factors might confound the relationship between recovery time and lung function improvement. Notably, 20–22% of our cohort continued to exhibit some form of pulmonary dysfunction 1 year after COVID‐19 infection, using either the 5th or the 2.5th percentile as the LLN (Table 1).

Our study has some limitations that should be considered. First, only 44 of the 82 patients had a HRCT scan for the final PFT. One reason for the missing HRCT scans is that patients needed to resume work, making follow‐up testing difficult. Second, the median length of time between PFT and CT scanning was 38 days. Logistically, it was difficult to schedule the HRCT scans at the same time as the PFT due to the lack of staffing and the fact that only one HRCT scanner was available. Third, there was heterogeneity in the timing of the two pulmonary evaluations, with one patient having only 67 days between evaluations and another having 637 days. Fourth, we were not able to systematically obtain haemoglobin measurements to correct DLCO, though all patients resided at sea level. Haemoglobin concentration does not usually improve model fit in reference equations (Stanojevic et al., 2017), so not having this information is of little concern. Finally, the absence of PFT results for prior COVID‐19 infection is a notable gap, though reference equations suggest a comparative impact on pulmonary function against a non‐affected cohort.

In conclusion, our study provides compelling evidence that nearly one‐quarter of patients with previous severe COVID‐19 still have pulmonary dysfunction approximately 1 year post‐diagnosis, with about 22% of patients showing abnormalities at a median of time of 1 year after contracting COVID‐19. The trajectory from abnormal to normal pulmonary function is individualized, with no association between the length of time to recover and the amount of improvement in pulmonary function. Nearly 30% of the variance in fibrosis scores from HRCT was shared with DLCO *z*‐scores, highlighting the complex nature of post‐COVID‐19 recovery and the need for comprehensive, multidisciplinary approaches to patient care. This research contributes to the growing body of knowledge on long‐term COVID‐19 outcomes and emphasizes the need for ongoing investigation into effective monitoring and treatment strategies for affected populations.

## AUTHOR CONTRIBUTIONS

Arturo Cortes‐Telles was responsible for the conception of the study, data acquisition, interpretation of the data, and revising the manuscript for important intellectual content. Luis Alberto Solís‐Díaz, Heidegger Mateos‐Toledo, and Jordan A. Guenette were responsible the interpretation of the data and revising the manuscript for important intellectual content. Gerald Stanley Zavorsky was responsible for the statistical analysis of the data, figures and table generation, interpretation of the data, writing the initial manuscript draft, and revising the manuscript for important intellectual content. All authors have read and approved the final version of this manuscript and agree to be accountable for all aspects of the work in ensuring that questions related to the accuracy or integrity of any part of the work are appropriately investigated and resolved. All persons designated as authors qualify for authorship, and all those who qualify for authorship are listed.

## CONFLICT OF INTEREST

None declared.

## FUNDING INFORMATION

None.

## Data Availability

The data that support the findings of this study are available on Mendeley Data, an online cloud repository for data (Zavorsky & Cortes‐Telles, 2024). As well, a further discussion of the dataset can be found in the following companion data article (Cortes‐Telles & Zavorsky, 2024).

## APPENDIX A

### How the smallest measurable change in summed *z*‐scores was determined

The summed *z*‐scores is the sum of the *z*‐scores for FEV_1_ + FVC + FEV_1_/FVC + DLCO + *V*_A_ + *K*_CO_. The *z*‐scores for FEV_1_, FVC and the ratio FEV_1_/FVC were obtained from race‐neutral spirometry reference equations (Bowerman et al., 2023). The *z*‐scores for DLCO, *V*_A_ and *K*_CO_ were obtained using Mexican Hispanic reference equations (Gochicoa‐Rangel et al., 2024). The day‐to‐day variability (SD or measurement error) in FEV_1_ and FVC is 130 and 170 mL, respectively (Lavin et al., 2015). This is converted to a day‐to‐day *z*‐score variability of 0.345 SD units for both, based on the spirometry reference equations (Bowerman et al., 2023). Given that the correlation between FEV_1_ and FVC from the current data is 0.976, then we can obtain the day‐to‐day variability in FEV_1_/FVC as:

$$\sqrt{{\sigma^{2}}_{\mathrm{FE}V_{1}}+{\sigma^{2}}_{\mathrm{FVC}}-2\;\times \;\rho_{\mathrm{FE}V_{1},\;\mathrm{FVC}}\times \sigma_{\mathrm{FVC}}}$$

where

${\sigma^{2}}_{\mathrm{FE}V_{1}}+{\sigma^{2}}_{\mathrm{FVC}}$ are the standard deviations squared of FEV_1_ (0.336^2^) and FVC (0.345^2^), respectively, and $\rho_{\mathrm{FEV}1,\mathrm{FVC}}$ is the correlation between FEV_1_ and FVC = 0.976.

Thus, the coefficient of variation for the FEV_1_/FVC ratio in *z*‐score units [CV(FEV_1_/FVC)] = 0.125 *z*‐score units, which is the day‐to‐day *z*‐score variability for FEV_1_/FVC.

The day‐to‐day variability (SD or measurement error) in DLCO and *V*_A_ is 1.6 mL/min/mmHg and 0.31 L, respectively (Lavin et al., 2015). This is converted to a day‐to‐day *z*‐score variability of 0.38 and 0.613 SD units, respectively, based on the Mexican Hispanic reference equations (Gochicoa‐Rangel et al., 2024). Given that the correlation between DLCO *z*‐scores and *V*_A_ *z*‐scores from the current data is 0.703, we can obtain the day‐today variability in *K*_CO_ as:

$$\sqrt{{\sigma^{2}}_{\mathrm{DLCO}}+{\sigma^{2}}_{\mathrm{VA}}-2\times \;\rho_{\mathrm{DLCO},\mathrm{VA}}\times \sigma_{\mathrm{DLCO}}\times \sigma_{\mathrm{VA}}}$$

where ${\sigma^{2}}_{\mathrm{DLCO}}+{\sigma^{2}}_{\mathrm{VA}}$ are the standard deviations squared of DLCO (0.38^2^) and *V*_A_ (0.613^2^), respectively, and $\rho_{\mathrm{DLCO},\mathrm{VA}}$ is the correlation between DLCO *z*‐scores and *V*_A_ *z*‐scores = 0.703. Thus, the coefficient of variation for *K*_CO_ in *z*‐score units (CV(*K*_CO_)) = 0.439 *z*‐score units = the day‐to‐day *z*‐score variability for *K*_CO_.

In summary, the day‐to‐day *z*‐score variability (*z*‐score measurement error) is for FEV_1_ = 0.345, FVC = 0.345, FEV_1/_FVC = 0.125, DLCO = 0.38, *V*_A_ = 0.613, and *K*_CO_ = 0.439 *z*‐score units. Thus, the day‐to‐day variability for the summed variances can now be calculated, taking into consideration that these *z*‐scores are correlated with each other.

Here are the following *z*‐score correlations from 82 subjects: FEV_1_ and FVC = 0.9760; FVC and DLCO = 0.577; DLCO and *K*_CO_ = 0.553; FEV_1_ and DLCO = 0.529; FVC and *V*_A_ = 0.812; *V*_A_ and *K*_CO_ = –0.100; FEV_1_ and *V*_A_ = 0.764; DLCO and *V*_A_ = 0.703; FEV_1_/FVC and DLCO = –0.254.

The variance of the sum of several correlated random variables can be calculated using the following formula:

$${\sigma^{2}}_{\mathrm{FE}V_{1}}+{\sigma^{2}}_{\mathrm{FVC}}+{\sigma^{2}}_{\mathrm{FE}V_{1}/\mathrm{FVC}}+{\sigma^{2}}_{\mathrm{DLCO}}+{\sigma^{2}}_{\mathrm{VA}}+{\sigma^{2}}_{\mathrm{KCO}}+2\times \Sigma \mathrm{Cov}\left(X_{i},X_{j}\right)$$

where $\Sigma \mathrm{Cov}(X_{i\;},X_{j})$ for each pair is defined as: $\Sigma \mathrm{Cov}\;\left(X_{i},X_{j}\right)=\rho x_{i},x_{j}\;\times \sigma x_{i}\times x_{j}$ where $\rho x_{i\;},x_{j}$ is the Pearson correlation coefficient between variables $X_{i\;}$ and $X_{j}$.

The calculation shows that the corrected day‐to‐day variability for the summed *z*‐scores is about 1.609 *z*‐score units. The difference between a subject's ‘summed’ measured *z*‐scores and the ‘summed’ true *z*‐scores would be expected to be less than 1.96 multiplied by the within‐subject SD (SD_w_) for 95% of observations, which is 2.77 SD_w_ (Bland & Altman, 1996), or 1.7746 × 2.77 = 4.457. When 4.457 is divided by 2, the smallest measurable change in summed *z*‐scores = 2.23. Taking the 95% limits of agreement (4.457) and dividing it by 2 (2.23) is more reasonable than using the 95% limits of agreement (Hopkins, 2000). A day‐to‐day change in summed *z*‐scores of more than ±2.23 units provides an 84% chance that this change in summed *z*‐scores, is, in fact, a true change (Hopkins, 2000).

## References

[eph13667-bib-0001] Abdallah, S. J. , Voduc, N. , Corrales‐Medina, V. F. , Mcguinty, M. , Pratt, A. , Chopra, A. , Law, A. , Garuba, H. A. , Thavorn, K. , Reid, R. E. R. , Lavoie, K. L. , Crawley, A. , Chirinos, J. A. , & Cowan, J. (2021). Symptoms, pulmonary function and functional capacity four months after COVID‐19. Annals of the American Thoracic Society, 18(11), 1912–1917. 33872135 10.1513/AnnalsATS.202012-1489RL PMC8641826

[eph13667-bib-0002] Angeles Montero‐Fernandez, M. , & Pardo‐Garcia, R. (2021). Histopathology features of the lung in COVID‐19 patients. Diagnostic Histopathology, 27(3), 123–127. 33312229 10.1016/j.mpdhp.2020.11.009 PMC7717771

[eph13667-bib-0003] Bellan, M. , Baricich, A. , Patrucco, F. , Zeppegno, P. , Gramaglia, C. , Balbo, P. E. , Carriero, A. , Amico, C. S. , Avanzi, G. C. , Barini, M. , Battaglia, M. , Bor, S. , Cantaluppi, V. , Cappellano, G. , Ceruti, F. , Chiocchetti, A. , Clivati, E. , Giordano, M. , Cuneo, D. , … Pirisi, M. (2021). Long‐term sequelae are highly prevalent one year after hospitalization for severe COVID‐19. Scientific Reports, 11(1), 22666. 34811387 10.1038/s41598-021-01215-4 PMC8608998

[eph13667-bib-0004] Bellan, M. , Soddu, D. , Balbo, P. E. , Baricich, A. , Zeppegno, P. , Avanzi, G. C. , Baldon, G. , Bartolomei, G. , Battaglia, M. , Battistini, S. , Binda, V. , Borg, M. , Cantaluppi, V. , Castello, L. M. , Clivati, E. , Cisari, C. , Costanzo, M. , Croce, A. , Cuneo, D. , … Pirisi, M. (2021). Respiratory and psychophysical sequelae among patients with COVID‐19 four months after hospital discharge. The Journal of The American Medical Association Network Open, 4(1), e2036142. 10.1001/jamanetworkopen.2020.36142 PMC7841464 33502487

[eph13667-bib-0005] Benjamini, Y. , & Yekutieli, D. (2001). The control of the false discovery rate in multiple testing under dependency. Annals of Statistics, 29(4), 1165–1188.

[eph13667-bib-0006] Blanco, J.‐R. , Cobos‐Ceballos, M.‐J. , Navarro, F. , Sanjoaquin, I. , Arnaiz De Las Revillas, F. , Bernal, E. , Buzon‐Martin, L. , Viribay, M. , Romero, L. , Espejo‐Perez, S. , Valencia, B. , Ibañez, D. , Ferrer‐Pargada, D. , Malia, D. , Gutierrez‐Herrero, F.‐G. , Olalla, J. , Jurado‐Gamez, B. , & Ugedo, J. (2021). Pulmonary long‐term consequences of COVID‐19 infections after hospital discharge. Clinical Microbiology and Infection, 27(6), 892–896. 33662544 10.1016/j.cmi.2021.02.019 PMC7920814

[eph13667-bib-0007] Bland, J. M. , & Altman, D. G. (1996). Measurement error. British Medical Journal, 312(7047), 1654. 8664723 10.1136/bmj.312.7047.1654 PMC2351401

[eph13667-bib-0008] Bowerman, C. , Bhakta, N. R. , Brazzale, D. , Cooper, B. R. , Cooper, J. , Gochicoa‐Rangel, L. , Haynes, J. , Kaminsky, D. A. , Lan, L. T. T. , Masekela, R. , Mccormack, M. C. , Steenbruggen, I. , & Stanojevic, S. (2023). A race‐neutral approach to the interpretation of lung function measurements. American Journal of Respiratory and Critical Care Medicine, 207(6), 768–774. 36383197 10.1164/rccm.202205-0963OC

[eph13667-bib-0009] Brems, J. H. , Balasubramanian, A. , Raju, S. , Putcha, N. , Fawzy, A. , Hansel, N. N. , Wise, R. A. , & Mccormack, M. C. (2024). Changes in spirometry interpretative strategies: Implications for classifying COPD and predicting exacerbations. Chest, 166(2), 294–303. 38537688 10.1016/j.chest.2024.03.034 PMC11317812

[eph13667-bib-0010] Chommeloux, J. , Valentin, S. , Winiszewski, H. , Adda, M. , Pineton De Chambrun, M. , Moyon, Q. , Mathian, A. , Capellier, G. , Guervilly, C. , Levy, B. , Jaquet, P. , Sonneville, R. , Voiriot, G. , Demoule, A. , Boussouar, S. , Painvin, B. , Lebreton, G. , Combes, A. , & Schmidt, M. (2023). One‐year mental and physical health assessment in survivors after extracorporeal membrane oxygenation for COVID‐19‐related acute respiratory distress syndrome. American Journal of Respiratory and Critical Care Medicine, 207(2), 150–159. 36150112 10.1164/rccm.202206-1145OC PMC9893333

[eph13667-bib-0011] Corsi, A. , Caroli, A. , Bonaffini, P. A. , Conti, C. , Arrigoni, A. , Mercanzin, E. , Imeri, G. , Anelli, M. , Balbi, M. , Pace, M. , Zanoletti, A. , Capelli, M. , Di Marco, F. , & Sironi, S. (2022). Structural and functional pulmonary assessment in severe COVID‐19 survivors at 12 months after discharge. Tomography, 8(5), 2588–2603. 36287815 10.3390/tomography8050216 PMC9611724

[eph13667-bib-0012] Cortes‐Telles, A. , Figueroa‐Hurtado, E. , Ortiz‐Farias, D. L. , & Zavorsky, G. S. (2022). Clinical predictors of lung function in patients recovering from mild COVID‐19. BMC Pulmonary Medicine, 22(1), 294. 35909118 10.1186/s12890-022-02086-9 PMC9339191

[eph13667-bib-0013] Cortés‐Telles, A. , López‐Romero, S. , Figueroa‐Hurtado, E. , Pou‐Aguilar, Y. N. , Wong, A. W. , Milne, K. M. , Ryerson, C. J. , & Guenette, J. A. (2021). Pulmonary function and functional capacity in COVID‐19 survivors with persistent dyspnoea. Respiratory Physiology & Neurobiology, 288, 103644. 33647535 10.1016/j.resp.2021.103644 PMC7910142

[eph13667-bib-0047] Cortes‐Telles, A. , & Zavorsky, G. S. (2024). Changes in spirometry and pulmonary diffusing capacity in Mexican Hispanics approximately one year after having severe COVID‐19: A dataset. *Data in Brief* . 10.1016/j.dib.2024.110998 PMC11541934 39512932

[eph13667-bib-0014] Ekbom, E. , Frithiof, R. , Emilsson, O. , Larson, L. M. , Lipcsey, M. , Rubertsson, S. , Wallin, E. , Janson, C. , & Hultström, M. (2021). Impaired diffusing capacity for carbon monoxide is common in critically ill Covid‐19 patients at four months post‐discharge. Respiratory Medicine, 182, 106394. 33901787 10.1016/j.rmed.2021.106394 PMC8047337

[eph13667-bib-0015] Fraser, E. , St Noble, V. , Hoyles, R. K. , Benamore, R. , & Ho, L. P. (2020). Readily accessible CT scoring method to quantify fibrosis in IPF. British Medical Journal Open Respiratory Research, 7(1), e000584. 10.1136/bmjresp-2020-000584 PMC7292044 32527873

[eph13667-bib-0016] Fumagalli, A. , Misuraca, C. , Bianchi, A. , Borsa, N. , Limonta, S. , Maggiolini, S. , Bonardi, D. R. , Corsonello, A. , Di Rosa, M. , Soraci, L. , Lattanzio, F. , & Colombo, D. (2022). Long‐term changes in pulmonary function among patients surviving to COVID‐19 pneumonia. Infection, 50(4), 1019–1022. 34652626 10.1007/s15010-021-01718-2 PMC8517554

[eph13667-bib-0017] Ghasemi, A. , & Zahediasl, S. (2012). Normality tests for statistical analysis: A guide for non‐statisticians. International Journal of Endocrinology and Metabolism, 10(2), 486–489. 23843808 10.5812/ijem.3505 PMC3693611

[eph13667-bib-0018] Gochicoa‐Rangel, L. , Hernández‐Morales, A. P. , Salles‐Rojas, A. , Madrid‐Mejía, W. , Guzmán‐Valderrábano, C. , González‐Molina, A. , Salas‐Escamilla, I. , Durán‐Cuellar, A. , Silva‐Cerón, M. , Hernández‐Morales, V. , Reyes‐García, A. , Alvarado‐Amador, I. , Lozano‐Martínez, L. , Enright, P. , Pensado‐Piedra, L. E. , & Torre‐Bouscoulet, L. (2021). Gas exchange impairment during COVID‐19 recovery. Respiratory Care, 66(10), 1610–1617. 34465571 10.4187/respcare.09114

[eph13667-bib-0019] Gochicoa‐Rangel, L. G. , De‐Los‐Santos Martinez, A. , Reyes‐Garcia, A. , Briseno, D. M. , Vargas, M. H. , & Lechuga‐Trejo, I. (2024). Reference equations for DLNO & DLCO in Mexican Hispanics: Influence of altitude and race. British Medical Journal Open Respiratory Research, 11(1), e002341. 10.1136/bmjresp-2024-002341 PMC11474816 39401975

[eph13667-bib-0020] Goh, N. S. L. , Desai, S. R. , Veeraraghavan, S. , Hansell, D. M. , Copley, S. J. , Maher, T. M. , Corte, T. J. , Sander, C. R. , Ratoff, J. , Devaraj, A. , Bozovic, G. , Denton, C. P. , Black, C. M. , Du Bois, R. M. , & Wells, A. U. (2008). Interstitial lung disease in systemic sclerosis: A simple staging system. American Journal of Respiratory and Critical Care Medicine, 177(11), 1248–1254. 18369202 10.1164/rccm.200706-877OC

[eph13667-bib-0021] Guler, S. A. , Ebner, L. , Aubry‐Beigelman, C. , Bridevaux, P.‐O. , Brutsche, M. , Clarenbach, C. , Garzoni, C. , Geiser, T. K. , Lenoir, A. , Mancinetti, M. , Naccini, B. , Ott, S. R. , Piquilloud, L. , Prella, M. , Que, Y.‐A. , Soccal, P. M. , Von Garnier, C. , & Funke‐Chambour, M. (2021). Pulmonary function and radiological features 4 months after COVID‐19: First results from the national prospective observational Swiss COVID‐19 lung study. European Respiratory Journal, 57(4), 2003690. 33419891 10.1183/13993003.03690-2020 PMC8082329

[eph13667-bib-0022] Han, X. , Fan, Y. , Alwalid, O. , Li, N. , Jia, X. , Yuan, M. , Li, Y. , Cao, Y. , Gu, J. , Wu, H. , & Shi, H. (2021). Six‐month follow‐up chest CT findings after severe COVID‐19 pneumonia. Radiology, 299(1), E177–E186. 33497317 10.1148/radiol.2021203153 PMC7841877

[eph13667-bib-0023] Hellemons, M. E. , Huijts, S. , Bek, L. M. , Berentschot, J. C. , Nakshbandi, G. , Schurink, C. A. M. , Vlake, J. H. , Van Genderen, M. E. , Van Bommel, J. , Gommers, D. , Odink, A. , Ciet, P. , Shamier, M. C. , Geurts Van Kessel, C. , Baart, S. J. , Ribbers, G. M. , Van Den Berg‐Emons, R. J. G. , Heijenbrok‐Kal, M. H. , & Aerts, J. G. J. V. (2022). Persistent health problems beyond pulmonary recovery up to 6 months after hospitalization for COVID‐19: A longitudinal study of respiratory, physical, and psychological outcomes. Annals of the American Thoracic Society, 19(4), 551–561. 34582728 10.1513/AnnalsATS.202103-340OC PMC8996273

[eph13667-bib-0024] Hopkins, W. G. (2000). Measures of reliability in sports medicine and science. Sports Medicine, 30(1), 1–15. 10907753 10.2165/00007256-200030010-00001

[eph13667-bib-0025] Huang, Y. , Tan, C. , Wu, J. , Chen, M. , Wang, Z. , Luo, L. , Zhou, X. , Liu, X. , Huang, X. , Yuan, S. , Chen, C. , Gao, F. , Huang, J. , Shan, H. , & Liu, J. (2020). Impact of coronavirus disease 2019 on pulmonary function in early convalescence phase. Respiratory Research, 21(1), 163. 32600344 10.1186/s12931-020-01429-6 PMC7323373

[eph13667-bib-0026] Konsberg, Y. , Szaro, P. , Aneman, A. , Kjellberg, S. , Solidakis, N. , Svedlund, S. , Nellgård, B. , & Dalla, K. (2023). Radiological appearance and lung function six months after invasive ventilation in ICU for COVID‐19 pneumonia: An observational follow‐up study. PLoS ONE, 18(9), e0289603. 37656699 10.1371/journal.pone.0289603 PMC10473523

[eph13667-bib-0027] Lavin, K. M. , Guenette, J. A. , Smoliga, J. M. , & Zavorsky, G. S. (2015). Controlled‐frequency breath swimming improves swimming performance and running economy. Scandinavian Journal of Medicine & Science in Sports, 25(1), 16–24. 10.1111/sms.12140 24151982

[eph13667-bib-0028] Liang, L. , Yang, B. , Jiang, N. , Fu, W. , He, X. , Zhou, Y. , Ma, W.‐L. , & Wang, X. (2020). Three‐month follow‐up study of survivors of Coronavirus disease 2019 after discharge. Journal of Korean Medical Science, 35(47), e418. 33289374 10.3346/jkms.2020.35.e418 PMC7721559

[eph13667-bib-0029] Liu, K. , Zhang, W. , Yang, Y. , Zhang, J. , Li, Y. , & Chen, Y. (2020). Respiratory rehabilitation in elderly patients with COVID‐19: A randomized controlled study. Complementary Therapies in Clinical Practice, 39, 101166. 32379637 10.1016/j.ctcp.2020.101166 PMC7118596

[eph13667-bib-0030] 3rd Mainous, A. G., , Rooks, B. J. , Wu, V. , & Orlando, F. A. (2021). COVID‐19 post‐acute sequelae among adults: 12 month mortality risk. Frontiers in Medicine, 8, 778434. 34926521 10.3389/fmed.2021.778434 PMC8671141

[eph13667-bib-0031] Mo, X. , Jian, W. , Su, Z. , Chen, M. , Peng, H. , Peng, P. , Lei, C. , Chen, R. , Zhong, N. , & Li, S. (2020). Abnormal pulmonary function in COVID‐19 patients at time of hospital discharge. European Respiratory Journal, 55(6), 2001217. 32381497 10.1183/13993003.01217-2020 PMC7236826

[eph13667-bib-0032] Morin, L. , Savale, L. , Pham, T. , Colle, R. , Figueiredo, S. , Harrois, A. , Gasnier, M. , Lecoq, A. L. , Meyrignac, O. , Noel, N. , Baudry, E. , Bellin, M. F. , Beurnier, A. , Choucha, W. , Corruble, E. , Dortet, L. , Hardy‐Leger, I. , Radiguer, F. , Sportouch, S. , … Monnet, X. (2021). Four‐month clinical status of a cohort of patients after hospitalization for COVID‐19. Journal of the American Medical Association, 325, 1525–1534. 33729425 10.1001/jama.2021.3331 PMC7970386

[eph13667-bib-0033] Qin, W. , Chen, S. , Zhang, Y. , Dong, F. , Zhang, Z. , Hu, B. , Zhu, Z. , Li, F. , Wang, X. , Wang, Y. , Zhen, K. , Wang, J. , Wan, Y. , Li, H. , Elalamy, I. , Li, C. , Zhai, Z. , & Wang, C. (2021). Diffusion capacity abnormalities for carbon monoxide in patients with COVID‐19 at 3‐month follow‐up. European Respiratory Journal, 58(1), 2003677. 33574077 10.1183/13993003.03677-2020 PMC7877322

[eph13667-bib-0034] Quanjer, P. H. , Stanojevic, S. , Cole, T. J. , Baur, X. , Hall, G. L. , Culver, B. H. , Enright, P. L. , Hankinson, J. L. , Ip, M. S. M. , Zheng, J. , & Stocks, J. (2012). Multi‐ethnic reference values for spirometry for the 3‐95‐yr age range: The global lung function 2012 equations. European Respiratory Journal, 40(6), 1324–1343. 22743675 10.1183/09031936.00080312 PMC3786581

[eph13667-bib-0035] Shah, A. S. , Wong, A. W. , Hague, C. J. , Murphy, D. T. , Johnston, J. C. , Ryerson, C. J. , & Carlsten, C. (2021). A prospective study of 12‐week respiratory outcomes in COVID‐19‐related hospitalisations. Thorax, 76(4), 402–404. 33273023 10.1136/thoraxjnl-2020-216308

[eph13667-bib-0036] Sonnweber, T. , Sahanic, S. , Pizzini, A. , Luger, A. , Schwabl, C. , Sonnweber, B. , Kurz, K. , Koppelstätter, S. , Haschka, D. , Petzer, V. , Boehm, A. , Aichner, M. , Tymoszuk, P. , Lener, D. , Theurl, M. , Lorsbach‐Köhler, A. , Tancevski, A. , Schapfl, A. , Schaber, M. , … Tancevski, I. (2021). Cardiopulmonary recovery after COVID‐19: An observational prospective multicentre trial. European Respiratory Journal, 57(4), 2003481. 33303539 10.1183/13993003.03481-2020 PMC7736754

[eph13667-bib-0037] Stanojevic, S. , Graham, B. L. , Cooper, B. G. , Thompson, B. R. , Carter, K. W. , Francis, R. W. , & Hall, G. L. (2017). Official ERS technical standards: Global Lung Function Initiative reference values for the carbon monoxide transfer factor for Caucasians. European Respiratory Journal, 50(3), 1700010. 28893868 10.1183/13993003.00010-2017

[eph13667-bib-0038] Stanojevic, S. , Kaminsky, D. A. , Miller, M. R. , Thompson, B. , Aliverti, A. , Barjaktarevic, I. , Cooper, B. G. , Culver, B. , Derom, E. , Hall, G. L. , Hallstrand, T. S. , Leuppi, J. D. , MacIntyre, N. , McCormack, M. , Rosenfeld, M. , & Swenson, E. R. (2022). ERS/ATS technical standard on interpretive strategies for routine lung function tests. European Respiratory Journal, 60(1), 2101499. 34949706 10.1183/13993003.01499-2021

[eph13667-bib-0039] Steinbeis, F. , Knape, P. , Mittermaier, M. , Helbig, E. T. , Tober‐Lau, P. , Thibeault, C. , Lippert, L. J. , Xiang, W. , Müller‐Plathe, M. , Steinbrecher, S. , Meyer, H.‐J. , Ring, R. M. , Ruwwe‐Glösenkamp, C. , Alius, F. , Li, Y. , Müller‐Redetzky, H. , Uhrig, A. , Lingscheid, T. , Grund, D. , … Zoller, T. (2022). Functional limitations 12 months after SARS‐CoV‐2 infection correlate with initial disease severity: An observational study of cardiopulmonary exercise capacity testing in COVID‐19 convalescents. Respiratory Medicine, 202, 106968. 36081267 10.1016/j.rmed.2022.106968 PMC9420203

[eph13667-bib-0040] Tarraso, J. , Safont, B. , Carbonell‐Asins, J. A. , Fernandez‐Fabrellas, E. , Sancho‐Chust, J. N. , Naval, E. , Amat, B. , Herrera, S. , Ros, J. A. , Soler‐Cataluña, J. J. , Rodriguez‐Portal, J. A. , Andreu, A. L. , Marín, M. , Rodriguez‐Hermosa, J. L. , Gonzalez‐Villaescusa, C. , Soriano, J. B. , Signes‐Costa, J. , García, Y. , Blasco, N. , … Vargas, G. (2022). Lung function and radiological findings 1 year after COVID‐19: A prospective follow‐up. Respiratory Research, 23(1), 242. 36096801 10.1186/s12931-022-02166-8 PMC9466319

[eph13667-bib-0041] Van Den Borst, B. , Peters, J. B. , Brink, M. , Schoon, Y. , Bleeker‐Rovers, C. P. , Schers, H. , Van Hees, H. W. H. , Van Helvoort, H. , Van Den Boogaard, M. , Van Der Hoeven, H. , Reijers, M. H. , Prokop, M. , Vercoulen, J. , & Van Den Heuvel, M. (2021). Comprehensive health assessment 3 months after recovery from Acute Coronavirus Disease 2019 (COVID‐19). Clinical Infectious Diseases, 73(5), e1089–e1098. 33220049 10.1093/cid/ciaa1750 PMC7717214

[eph13667-bib-0042] WHO . (2023). Clinical management of COVID‐19: Living guideline, 18 August 2023 (pp. 1–186) Geneva. (WHO/2019‐nCoV/clinical/2023.2). License: CC BY‐NC‐SA 3.0 IGO. World Health Organization (WHO).

[eph13667-bib-0043] Wong, A. W. , López‐Romero, S. , Figueroa‐Hurtado, E. , Vazquez‐Lopez, S. , Milne, K. M. , Ryerson, C. J. , Guenette, J. A. , & Cortés‐Telles, A. (2021). Predictors of reduced 6‐minute walk distance after COVID‐19: A cohort study in Mexico. Pulmonology, 27(6), 563–565. 33832849 10.1016/j.pulmoe.2021.03.004 PMC7997705

[eph13667-bib-0044] Wu, W. J. , Huang, W. M. , Liang, C. H. , & Yun, C. H. (2022). Pulmonary vascular volume is associated with DLCO and fibrotic score in idiopathic pulmonary fibrosis: An observational study. BioMed Central Medical Imaging, 22(1), 76. 35461214 10.1186/s12880-022-00803-8 PMC9034618

[eph13667-bib-0045] Wu, X. , Liu, X. , Zhou, Y. , Yu, H. , Li, R. , Zhan, Q. , Ni, F. , Fang, S. , Lu, Y. , Ding, X. , Liu, H. , Ewing, R. M. , Jones, M. G. , Hu, Y. , Nie, H. , & Wang, Y. (2021). 3‐month, 6‐month, 9‐month, and 12‐month respiratory outcomes in patients following COVID‐19‐related hospitalisation: A prospective study. Lancet Respiratory Medicine, 9(7), 747–754. 33964245 10.1016/S2213-2600(21)00174-0 PMC8099316

[eph13667-bib-0046] Zavorsky, G. S. , & Cao, J. (2022). Reference equations for pulmonary diffusing capacity using segmented regression show similar predictive accuracy as GAMLSS models. British Medical Journal Open Respiratory Research, 9(1), e001087. 10.1136/bmjresp-2021-001087 PMC8852756 35172984

[eph13667-bib-0048] Zavorsky, G. S. , & Cortes‐Telles, A. (2024). Long‐term changes in spirometry and diffusing capacity in Mexican Hispanics with previous severe COVID‐19, Mendeley Data, V4. 10.17632/xmmyb9rgjj.4 PMC11541934 39512932

[eph13667-bib-0049] Zhao, Y. , Yang, C. , An, X. , Xiong, Y. , Shang, Y. , He, J. , Qiu, Y. , Zhang, N. , Huang, L. , Jia, J. , Xu, Q. , Zhang, L. , Zhao, J. , Pei, G. , Luo, H. , Wang, J. , Li, Q. , Gao, Y. , & Xu, A. (2021). Follow‐up study on COVID‐19 survivors one year after discharge from hospital. International Journal of Infectious Diseases, 112, 173–182. 34520845 10.1016/j.ijid.2021.09.017 PMC8434916

[eph13667-bib-0050] Zhao, Y.‐M. , Shang, Y.‐M. , Song, W.‐B. , Li, Q.‐Q. , Xie, H. , Xu, Q.‐F. , Jia, J.‐L. , Li, L.‐M. , Mao, H.‐L. , Zhou, X.‐M. , Luo, H. , Gao, Y.‐F. , & Xu, A.‐G. (2020). Follow‐up study of the pulmonary function and related physiological characteristics of COVID‐19 survivors three months after recovery. eClinicalMedicine, 25, 100463. 32838236 10.1016/j.eclinm.2020.100463 PMC7361108

[eph13667-bib-0051] Zhou, F. , Tao, M. , Shang, L. , Liu, Y. , Pan, G. , Jin, Y. , Wang, L. , Hu, S. , Li, J. , Zhang, M. , Fu, Y. , & Yang, S. (2021). Assessment of sequelae of COVID‐19 nearly 1 year after diagnosis. Frontiers in Medicine, 8, 717194. 34888318 10.3389/fmed.2021.717194 PMC8649686

